# Feline Foamy Virus-Based Vectors: Advantages of an Authentic Animal Model

**DOI:** 10.3390/v5071702

**Published:** 2013-07-12

**Authors:** Weibin Liu, Janet Lei, Yang Liu, Dragana Slavkovic Lukic, Ann-Mareen Räthe, Qiuying Bao, Timo Kehl, Anne Bleiholder, Torsten Hechler, Martin Löchelt

**Affiliations:** Department of Genome Modifications, Research Program Infection and Cancer, German Cancer Research Center, Im Neuenheimer Feld 242, 69120 Heidelberg, Germany; E-Mails: liu.weibin@mayo.edu (W.L.); janet.lei@dkfz.de (J.L.); y.liu@dkfz.de (Y.L.); d.slavkovic@dkfz.de (D.S.L.); a.raethe@dkfz.de (A.-M.R.); q.bao@dkfz.de (Q.B.); t.kehl@dkfz.de (T.K.); a.bleiholder@dkfz.de (A.B.); t.hechler@hdpharma.com (T.H.)

**Keywords:** retrovirus vector, foamy virus, vaccine vector, gene therapy vector, animal model, spumaretrovirus, replication-deficient vector, replication-competent vector

## Abstract

New-generation retroviral vectors have potential applications in vaccination and gene therapy. Foamy viruses are particularly interesting as vectors, because they are not associated to any disease. Vector research is mainly based on primate foamy viruses (PFV), but cats are an alternative animal model, due to their smaller size and the existence of a cognate feline foamy virus (FFV). The potential of replication-competent (RC) FFV vectors for vaccination and replication-deficient (RD) FFV-based vectors for gene delivery purposes has been studied over the past years. In this review, the key achievements and functional evaluation of the existing vectors from *in vitro* cell culture systems to out-bred cats will be described. The data presented here demonstrate the broad application spectrum of FFV-based vectors, especially in pathogen-specific prophylactic and therapeutic vaccination using RD vectors in cats and in classical gene delivery. In the cat-based system, FFV-based vectors provide an advantageous platform to evaluate and optimize the applicability, efficacy and safety of foamy virus (FV) vectors, especially the understudied aspect of FV cell and organ tropism.

## 1. Introduction

The use of viral vectors in clinical therapy is an emerging field. In addition to the established methods of generating viral vaccines from attenuated or inactivated virus or viral derivatives, viruses, especially retroviruses, can be used as a vector for gene-mediated therapy. In 2012, an HIV-based vector was used to transduce T-cells with a chimeric antigen receptor *ex vivo* against acute lymphoid leukemia [1]. Additional potential retroviral vectors are other lentiviruses, gammaretroviruses, such as murine leukemia virus, and foamy viruses (FVs). In particular, FVs are not associated to any diseases and are therefore of interest for safe retroviral vaccine development.

FV vectors have been successfully applied in several veterinary settings. Dogs suffering from canine leukocyte adhesion deficiency were treated with a recombinant FV expressing CD18 [2]. Long‑term follow-up showed that the animals enjoyed more than four years of disease-free survival [3]. In addition to gene therapy, FVs are also used as antigen carriers in recombinant vaccines. Cats infected with feline calicivirus were treated with feline foamy virus (FFV) replication-competent (RC) vectors displaying capsid protein E domain epitopes, which led to partial suppression of clinical feline calicivirus (FCV) symptoms [4].

Of course, there are, as with other retroviruses, concerns that insertional mutagenesis, due to FV integration into the genome, which may have a pathological effect and potentially lead to the development of cancer or other genetic irregularities. However, a number of studies on retroviral integration sites [5,6,7] indicate that FVs are far less likely to integrate into transcriptionally active regions when compared to classical retroviral vectors, such as lentiviruses.

Retroviral vectors based on the genome of the prototype or primate foamy virus (PFV, formerly also designated human foamy virus, HFV) have been shown to possess several advantageous features for vector-based targeted gene transfer [8,9,10]. In fact, the majority of FV vectors described in the literature are derived from PFV and a few of the related simian FVs (SFVs). Due to the genetic relatedness of the original simian/primate/ape hosts to human beings, PFV- and SFV-derived vectors have the advantage that they efficiently transduce several human cell lines, a feature not met to this degree by FFV-derived vectors when using diverse human cells and cell lines [11]. 

There is no reliable and/or fully or at least partially permissive small animal model for PFV replication, although very low levels of replication may be detectable in PFV-infected mice and SFV‑infected hamster [12,13]. This necessitates the use of primates for functional studies of PFV‑based replicating vectors for vaccination purposes. Such studies using RC FV vectors in primates are often either not possible at all or extremely costly, allowing only small-scale studies. 

PFV has a broad tropism *in vitro*, though therapeutic gene transduction into a simian or a cat animal model using PFV has never been attempted. Due to extensive passaging in cell culture, PFV has accumulated a number of mutations that allow promiscuous infection of cell lines and may not reflect the authentic virus from primates. Studies of PFV in a mouse model have also been described [12]. Although the animals seroconverted and PFV DNA was detected by PCR, no virus re-isolation was reported, leaving it open whether the virus had been able to induce a productive, full-blown infection. Related studies in a hamster model have yielded similar results [13]. In both these studies, the PFV vectors were applied *ex vivo*. 

The isolate used as backbone for the current PFV vectors is the end-product of the zoonotic transmission of a chimpanzee SFV to humans [8,14,15,16]. This single PFV isolate had suffered substantial deletions in the viral long terminal repeat (LTR) sequences, and the PFV Bet protein also appears less functional in protecting the virus against host-mediated APOBEC3 restriction than its FFV counterpart [17,18,19,20]. Both features are likely the consequence of prolonged propagation of PFV in diverse human and non‑human cell lines. Therefore, PFV and PFV-derived vectors may have an attenuated replication capacity through suboptimal counteraction of antiviral activities in humans and non-human primates. In contrast, molecular clones of FFV have been shown to be fully RC in cats [4,21,22].

Due to stifling regulations on primate experimentation, gene therapy in non-human primates (NHPs) has not been attempted with PFV/SFV vectors. Although there are also public concerns regarding feline experimentation, cats are more easily accessible for statistically significant FFV vector studies. Experimental infections of cats with molecularly cloned and/or engineered FFV genomes and vectors have been previously described by us and others [4,21,22,23].

Because there is no cognate replication-competent mouse or other small laboratory animal FV known, the cat is the smallest established animal model where the cognate FV is replication-competent. In addition to the ability to host a persistent infection, transgenic tools for cats have been recently developed, which will prove to be a great advantage in modeling human diseases in cats [24]. Furthermore, as potential veterinary vaccines against viral cat pathogens, FFV has been used in proof‑of-concept studies for vectors that may ultimately be used in human therapy [4]. Finally, cats are already widely used in HIV studies, where clinical symptoms are better modeled during feline immunodeficiency virus (FIV) infections than simian immunodeficiency virus (SIV) infections in primates [25].

Only a few groups are currently working on FFV and FFV vectors. Several RC FFV genomes from both known FFV serotypes have been constructed [23,26,27]. Importantly, infection with either serotype does not appear to induce cross-neutralizing antibodies, a feature highly advantageous for repeated FFV vector applications. While the neutralization specificity of both known FFV serotypes is related to differences in the surface (SU) domain of the Env viral envelope glycoprotein [27,28], the basal molecular biology and features important for vector function are expected to be highly conserved. This implies that FFV vector backbones are interchangeable, except for the surface (SU) ectodomain and that functional data for one serotype are most likely also valid for the other. 

**Figure 1:**
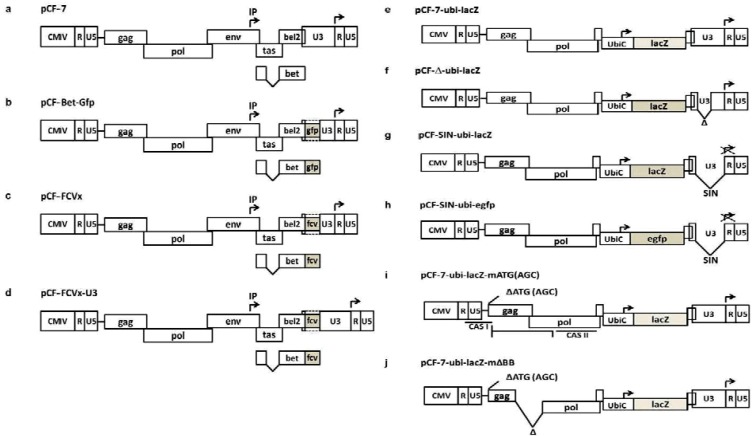
Schematic presentation of the different replication-components (RCs) (**a**–**d**, left panel) and replication-deficient (RD) (**e**–**j**, right panel) FFV vectors developed in the lab of the authors. FFV genes and long terminal repeats (LTRs) (U5, R, U3 regions) and heterologous genes are represented by boxes (not to scale). Broken arrows mark promoters and direction of transcription; inactivated promoters, by crosses; and deleted sequences and splice variants, as broken lines. (**a**) Authentic RC and CMV-IE promoter-driven FFV vector pCF-7 [22]. (**b**) Parental green fluorescent protein (GFP) expression vector pCF-Bet-Gfp. *gfp* is shown as a shaded box inserted into the deletion in the U3 region of the 3' LTR [22]. (**c**) Hybrid FFV-FCV clones (pCF-FCVx) carrying a deletion in the U3 of the 3' LTR. The FCV E inserts (E14, E23, and E24) are represented by shaded boxes [4]. (**d**) Chimeric FFV-FCV clones (pCF-FCVx-U3) with reconstructed U3. The different FCV E inserts (E14, E23, and E24) are represented by shaded boxes [4]. (**e**–**g**) The ubi-lacZ vectors, pCF‑7-ubi-lacZ, pCF-Δ-ubi-lacZ and pCF-SIN-ubi-lacZ, with the intact, truncated and functionally deleted Self-inactivating (SIN) LTR promoter and the ubi-lacZ cassette [30]. (**h**) pCF-SIN-ubi-egfp with *egfp* inserted into pCF-SIN-ubi-lacZ vectors by replacing *lacZ* [54]. (**i**–**j**) ubi-lacZ vectors pCF-7-ubi-lacZ-mATG (AGC) with mutagenesis of the *gag* ATG and pCF-7-ubi-lacZ-mΔBB with mutagenesis of the gag ATG and truncated *gag-pol* [54].

In cats, FFV actively infects and is released from cells of the oral mucosa [21]. Immunohistochemistry of tissues isolated from experimentally infected cats confirmed the presence of FFV in peripheral white blood cells, including macrophages and fibroblasts isolated from intestinal connective tissue [29]. In addition to productive infections in cats *in vivo*, FFV can also transduce human and other animal cell lines, albeit at a low level, and did not transduce mouse cell lines [30]. Zoonotic transmission of FFV into humans seems unlikely [28,31]. Conversely, PFV was shown to latently infect peripheral blood lymphocytes [32], but have never been tested on cat cell lines. In this review, we will describe the efforts related to the construction of full-length and infectious FFV genomes and the features and perspectives of currently-used RC and replication-deficient (RD) FFV-based vectors (as summarized in Figure 1 and Table 1, Table 2).

**Table 1:** Selected replication-competent feline foamy virus (FFV) vectors and their properties. HFV, human foamy virus; PFV, primate foamy virus; CMV-IE, cytomegalovirus immediate-early.

Vector	Virus species	Reference	Characteristics
SKY4.0	FFV/HFV	[23]	Hybrid FFV genome with PFV env and bel1/tas replacing the corresponding FFV sequences
pChatul-2/3	FFV	[26]	wt FFV-F17 genomic vector driven by the CMV-IE promoter instead of the FFV U3 promoter
pCF-7	FFV	[22]	wt FFV-FUV genomic vector driven by the CMV-IE promoter instead of the FFV U3 promoter
pCF-Bet-Gfp	FFV	[22]	Gfp marker gene expressed as a Bet-Gfp fusion protein or via an IRES element
pCF-IRES-Gfp			
pCF-FCVx	FFV/FCV	[4]	Recombinant FFV vaccine vectors expressing FCV capsid epitopes as Bet-FCV-E fusion proteins
pCF-FVCx-U3			

## 2. Construction of Full-Length and Replication-Competent FFV Genomes

Due to technological limitations related to full-length PCR amplification and cloning of intact FV genomes at the end of the last century, the FFV genomes of the FUV [28], F17 [33], Coleman, and S7801 isolates [23] were initially cloned in overlapping, sub-genomic fragments and subsequently assembled into full-length FFV genomes [22,23,26,27,34]. The replication competence of the resulting proviruses was assayed in transfected cells. For some of the original FFV full-length clones, Env sequences were replaced [27], or new variants were selected in cell culture and re-cloned [26], to gain full replication-competence upon plasmid DNA transfection into permissive feline cells. 

The promoter in the 5'-LTR in the FUV and F17 FFV vector genomes was replaced by the strong and constitutively active human cytomegalovirus immediate-early (CMV-IE) promoter [4,22,26] to increase vector titers and to generate progeny virus independent of the FFV Tas/Bel1 transactivator of spumaviruses (Table 1 and Figure 1a). This manipulation also allows easier engineering of the recombinant genomes, since the U3 region is no longer repeated at either end of the genome. 

Replication of the progeny virus was assayed in experimentally inoculated cats for the CMV-IE-driven pCF-7 genome of the FFV FUV genotype and the FFV LTR-driven Coleman isolate [22,23]. Replication of the Coleman isolate was shown by FFV Gag seroconversion and detection of FFV DNA in peripheral blood leukocytes by PCR at 30 d p.i. [23]. The replication of the pCF-7-derived FFV‑FUV was analyzed in detail with respect to virus re-isolation and broad-spectrum reactivity against FFV antigens: the cloned virus was found to be undistinguishable from the parental FUV isolate [21], demonstrating that the cloned FFV FUV genome can be used as a starting point for the construction of replication competent and deficient FFV vectors [22].

## 3. Replication-Competent FFV Gene Transfer Vectors

An FFV Coleman-based proviral DNA clone, containing almost the entire PFV *env* and the N‑terminal part of PFV *bel1/tas* instead of the corresponding FFV sequences, was constructed and analyzed *in vitro* and *in vivo* [23]. In this hybrid genome, the viral transcriptional transactivator Bel1/Tas is composed of PFV and FFV regions. Two evolutionary unrelated promoters are present in this genome: the FFV Bel1/Tas-specific LTR promoter and the PFV Bel1/Tas-specific internal promoter found in the swapped PFV *env* gene (Table 1). This clone was reported to be RC in cell culture and cats, though other groups have published data on the species-specificity of the Bel1/Tas proteins and the internal promoters of FFV and PFV [35,36,37,38]. Additional supporting data, such as a quantitative evaluation of the replication competence of the FFV-PFV chimera, have not been presented [23]. Due to its chimeric nature, the replication ability of this vector is most likely highly attenuated.

In our lab, we also engineered RC FFV vectors for gene and antigen transfer. Since no function had been attributed to FV *bet* at the time, we initially attempted to replace the central and 3' part of FFV *bet*, which does not overlap with the essential *bel1/tas* transactivator gene by heterologous reporter genes. Bet was chosen as a carrier to express heterologous proteins, since it is highly expressed *in vitro* and *in vivo*, as reflected by the presence of Bet-specific antibodies in naturally or experimentally FV‑infected animals [21,29]. In addition, FV Bet/Bel2-Env fusion proteins or Bet proteins that are actively secreted or specifically released from infected cells in Env-dependent and -independent fashions can even be taken up by other cells [39,40,41,42]. In order to facilitate insertion of foreign sequences into *bet*, a multiple cloning site was placed downstream of *bel1/tas* [43]. Surprisingly, the replication capacity of the resulting vectors was severely (up to 1,000-fold) attenuated in FFV‑permissive CRFK cells, indicating that *bet* is required, even *in vitro* [43]. Only years later, after identification of APOBEC3-mediated restriction of HIV and the importance of HIV Vif protein in counteracting this antiviral activity [44], we and others found that FV Bet proteins serve similar functions for FVs by counteracting APOBEC3 restriction of feline and primate hosts [18,19,45,46,47,48].

Due to the essential nature of Bet, we then aimed to attach heterologous sequences to the 3'-end of *bet*, located in the U3 part of the 3' long terminal repeat (LTR, Figure 1b). This approach was successful, allowing the recovery of engineered, fully RC vectors efficiently expressing green fluorescence protein (GFP) [22]. Importantly, these vectors lacked FFV U3 regions from −725 to −308 relative to the transcriptional start site. However, upon serial passaging of these RC FFV vectors, the heterologous *gfp* sequences were successively lost, and GFP marker gene transfer ceased after about ten passages [22]. Although the vectors displayed only limited genetic stability, the concept of FFV‑based RC vectors was experimentally validated.

## 4. Replication-Competent FFV Vaccine Vectors

Based on the data described above, we constructed RC FFV vectors for cat immunization against an acute and severe disease for which an efficient prophylactic vaccine was not yet available. Especially for the latter reason, the feline calicivirus (FCV) model was selected, as FCV induces severe disease in adult and often fatal disease in juvenile cats [49,50,51,52]. However, at the time when the project was started, none of the FCV E1 neutralizing antibody-inducing epitopes had been fully characterized [49,50,51,52,53]. To reduce the risk of losing the inserted FCV epitopes, FCV E1 sequences of only 39, 106 and 146 aa were fused to the C-terminus of FFV Bet as the vaccine antigen [4]. The FCV E1 sequence with a length of 106 aa displayed reasonable genetic stability in the FFV background and was thus selected as insert. Since it was not clear whether an intact LTR is required for vector replication and gene expression, replicating FFV-FCV vectors with a U3 containing a partially deleted LTR (Figure 1c) and an intact LTR (Figure 1d) were analyzed in cats. 

After a single injection of the FFV vaccine vectors, no treatment-related complications were evident in any of the vaccinated cats. Clear seroconversion against the FFV vectors was detectable in all treated cats. Most of the cats vaccinated with the FCV-FFV vector carrying the reconstructed LTR promoter (Figure 1d) displayed E1 epitope-specific antibodies, which were low or absent in animals that received the U3-deleted FCV E1 vector (Figure 1c). None of the vaccinated animals had acquired detectable levels of FCV-neutralizing antibody titers. Vector recovery was possible for some of the U3-reconstructed vector-treated or empty vector control animals [4]. 

As determined after intranasal challenge with a high dose of pathogenic FCV, none of the FCV E1 vaccinated cats had developed sterile immunity. However, partial protection was achieved by the FFV‑based vaccination, as evidenced by a drastic reduction of FCV-induced disease symptoms and reduced FCV shedding. As expected from the lack of neutralizing antibodies before challenge, the vaccination effect was not correlated to E1-binding antibodies. Instead, it appeared that animals that did not mount FCV-specific seroreactivity were especially protected from clinical symptoms, such as the development of oral and nasal lesions and ulcers. Symptom manifestation also correlated with a deletion in the U3 promoter region, which was suggested to be a contributing factor to increased genetic stability of the hybrid vectors [4]. 

In summary, the data demonstrate the applicability and potential of FFV-based vaccine vectors to (partially) induce protective immunity against a pathogenic FCV challenge infection. In addition, the data suggest that the protection achieved was mediated by cellular immune mechanisms that were, unfortunately, not further studied or specified [4]. Depending on the replicative capacity, RC FFV titers of more than 10^7^ transducing units/mL unconcentrated supernatant can be achieved. In addition, RC vectors can be easily amplified in Crandall-Rees feline kidney CRFK cell cultures. Passage through feline cells also induces feline-specific protein modification and prevents vector rejection by unspecific immunity in cats against human proteins that would be relevant for human 293T cell‑derived vectors. 

## 5. Replication-Deficient FFV Vectors

Similar to first-generation RD PFV vectors, [55,56], FFV serotype F17-based RD vectors driven by the heterologous CMV-IE promoter and with a heterologous promoter-marker gene cassette replacing the *env-bel* gene region were constructed and used in studies related to the timing of reverse transcription in FVs and the restriction of FV replication by cellular TRIM5α proteins [26,57].

**Table 2:** Selected replication-deficient FFV vectors and their properties.

Vector	Reference	Description
pCF-SIN pCF-Bet-Gfp-SIN	[58]	Self-inactivating (SIN) FFV Bet-Gfp vectors
pCF-7-ubi-lacZ pCF-Δ-ubi-lacZ pCF-SIN-ubi-lacZ	[30]	*Env*- and *bel*-deficient FFV vectors expressing the lacZ gene from the ubiC promoter and with intact, truncated or SIN LTR promoters
pCF-7-ubi-lacZ-mATG pCF-7-ubi-lacZ-mΔBB	[54]	pCF^−/−^ubi-lacZ-derived vectors with inactivated gag ATG start codon and gag-pol deletions
pCF-SIN-ubi-egfp	described here	pCF-SIN-ubi-lacZ-derived vectors in which the detrimental lacZ reporter gene was replaced by the neutral egfp gene

### 5.1. First-Generation RD FFV Vectors

The development of the FFV serotype replication-deficient FUV vectors is summarized in Table 2 and depicted in Figure 1e–j. As an alternative approach to RC vectors, self-inactivating (SIN) vectors were constructed by deleting the 3' LTR promoter (from –725 to –18 relative to the transcriptional start site). The resultant SIN progeny should undergo a single round of replication, as the proviral genome, upon reverse transcription and LTR formation, is devoid of functional promoters on both sides of the SIN-LTR [58]. These CMV-IE-driven FFV genomes lacking the LTR promoter and encoding either WT Bet or a Bet-Gfp fusion protein (see above) were shown to efficiently express Bet or Bet-Gfp (Table 2). The genetic stability of FFV SIN Bet-Gfp construct and incidence of reversion in cell culture was assessed. Upon serial passages, replication-competent revertants (RCRs) containing different, but apparently functional, LTR to CMV-IE replacements were detected. Incorporation of CMV-IE fragments of variable sizes into the SIN-deleted promoter resulted in attenuated RCRs with substantial virus titers [58]. These recombination events were probably favored by sequence homologies between the 5' (CMV-IE-R-U5) and the 3' (SIN-R-U5) LTRs in the genome. These data clearly showed that even substantial SIN deletions are not sufficient to abrogate RCR generation.

### 5.2. Second-Generation RD FFV Vectors

Second-generation RD vectors were constructed from LTR SIN mutants (Table 2 and Figure 1e–g) with different deletion-replacement combinations of the *env-bel* region by a ubiquitin C promoter (ubiC)-lacZ cassette (ubi-lacZ) [30]. The combined self-inactivation and *env-bel* deletion yielded a highly advantageous safety profile, even when transduced cells were super-infected with WT FFV, a situation favoring RCR generation via recombination between FFV-derived vector components and the WT FFV genome. Vectors with partially deleted LTR promoters, as described for FFV vaccines, were only slightly less efficient than those with the WT LTR. Although the safety profile of the vectors was excellent, a slight reduction of the number of transduced cells became detectable over time. This could be due to detrimental effects related to lacZ expression or to the inactivation of vector-encoded gene expression, for instance, by epigenetic mechanisms, like DNA or histone methylation [30]. CpG dinucleotides are the predominant target sequences for cytosine methylation in vertebrate genomes [59]. CpG plot analysis (European Molecular Biology Laboratory-European Bioinformatics Institute, EMBL-EBI) revealed a low-CpG content in the FFV-vector sequences, advantageous for the prevention of methylation-mediated gene silencing. In contrast, CpG is abundant in the ubi-lacZ cassette. DNA methylase inhibitor treatment of transduced cells slightly increased the LacZ+ cell population [11]. 

**Figure 2:**
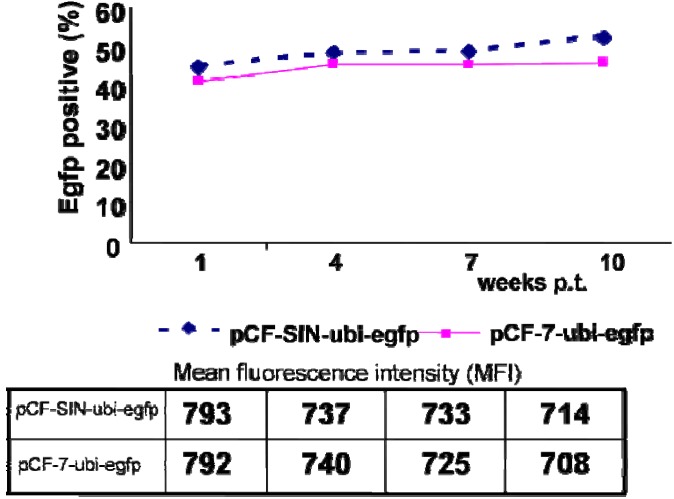
Stable *egfp* expression in ubi-egfp vector-transduced Crandall-Rees feline kidney (CRFK) cells. Stability of transgene expression is expressed as the percentage of Egfp‑positive cells (top panel) and the mean fluorescence intensity (MFI, bottom panel). CRFK cells were transduced with pCF-SIN-ubi-egfp and pCF-7-ubi-egfp at a multiplicity of infection (MOI) of 0.5. Transduced cells were passaged twice weekly and analyzed by flow cytometry every three weeks post-transduction. Quantitative results are given by the percentage of Egfp-positive cells and the MFI as a marker for the expression level, according to each flow cytometry assay.

To assess the effect of different marker genes on FFV vector-mediated transgene stability, lacZ was replaced by the eukaryotic and neutral egfp gene (Figure 1h), which encodes the enhanced green fluorescence protein (EGFP) and has a similar CpG content as the lacZ gene, in two different vectors harboring WT LTR or SIN LTR. The stability of the ubi-egfp transgene in FFV RD vectors was monitored by flow cytometry every three weeks for up to 10 weeks post-transduction. As shown in Figure 2, the percentage of EGFP-positive cells remained stable in both pCF-SIN-ubi-egfp and pCF-7-ubi-egfp transduced cells (Figure 2, top panel), while the mean EGFP fluorescence intensity decreased slightly in the long-term (Figure 2, bottom panel). These data suggest that decreased LacZ expression by ubi-lacZ-transduced cells is due to the lacZ transgene itself, rather than the FFV RD vector backbone. Therefore, second-generation FFV RD vectors allow stable transgene expression, though stability may be influenced by the choice of the transgene. 

### 5.3. Third-Generation Gutless FFV Vectors

To optimize the insertion capacity of the FFV vectors and to further increase vector safety, third-generation gutless FFV vectors devoid of any functional FFV genes were designed, constructed and characterized (Table 2 and Figure 1i,j). The identification of essential cis-elements was performed using the RC FFV Bet-Gfp vector [60] or the RD env-bel-deleted ubi-lacZ vector [54]. These studies were guided in part by corresponding studies in PFV-based vectors and molecular biology studies on PFV and FFV, especially those related to genome packaging and Gag and Pol protein expression [14,28,61]. As in orthoretroviral and PFV-derived vectors, FFV sequences from the cap site to the 5'‑part of gag were expected to contain the primer binding site (PBS), the dimer linkage site (DLS), the RNA packaging site (Psi) and the Pol encapsidation signal I (PESI) [62,63,64]. These sequences are collectively designated FV *cis*-acting sequences I (CASI), which are absolutely essential for vector function [64]. Studies on PFV showed that a second CAS (CASII) is located in pol [62,63,64].

To increase vector safety and avoid the generation of LTR-derived transcripts, which are fused via splicing to adjacent cellular sequences upon provirus integration, the major 5' splice donor (5'SD) located in the R region of the FFV 5'-LTR was inactivated by site-specific random mutagenesis. None of the tested 5'SD mutants supported vector function, and even compensatory mutations aimed at restoring leader RNA folding were non-functional when tested for marker gene transduction [54]. It was found that the intact 5'SD is required for the cytoplasmatic accumulation of FFV-based RNA vector genomes [54]. Similarly, deletion of the 3'-end of the 5'-untranslated FFV leader sequence was not compatible with vector function [60]. 

There is currently no satisfying explanation for the finding that even minimal mutagenesis at and around the FFV gag start codon drastically decreases FFV vector titers [54,60]. However, random mutagenesis and functional analysis of individual vector genomes allowed identification of a single vector genome with a completely abrogated Gag protein expression via inactivation of the start codon (ATG to AGC) that displayed almost full vector genome function [54]. The inactivation of the gag start codon also circumvents problems related to truncated Gag or artificial Gag-Pol fusion proteins that were shown before to negatively affect FFV vector titers [60]. Thus, gutless FFV vectors should contain the ATG to AGC gag start codon mutation. In summary, the localization of CASI functions identified for PFV is conserved in the FFV genome (see Figure 1i). 

Additional deletions affecting the *gag* and *pol* coding sequences were introduced, showing that *gag* is not essential to the vector, while the loss of central and 3'-terminal pol sequences abrogated vector function [54,60]. In particular, a 1.9 kb FFV *pol*-derived fragment that does not overlap with *env* sequences allowed full vector function, whereas the removal of an additional 550 bp resulted in a 10‑fold decrease in marker gene transfer [54]. As in PFV, this pol-derived FFV sequence should function as the Pol encapsidation signal [65,66] contained within CASII. Though FFV CASII does not extend into *env*, the overall organization of the essential cis-acting elements for vector function is conserved between PFV and FFV. 

During construction of the FV packaging plasmids, it was shown [54,60] that FFV Gag, like PFV, requires intact splice sites or the Woodchuck Hepatitis Virus post-transcriptional regulatory element (WPRE) for efficient gene expression when taken out of the proviral context [54,67]. In contrast, FFV Pol and Env expression does not require such elements, indicating that a negative-regulatory element is located within *gag*. Depending on the genetic outfit and the deletions and substitutions introduced, FFV RD vector titers of more than 10^5^ transducing units/mL of unconcentrated supernatant can be in general achieved. 

Pseudotyping is an extremely useful tool in virus molecular biology and has particularly interesting applications in clinical therapy. Due to their divergent evolution from the orthoretroviruses, however, direct pseudotyping of FFV vectors with diverse heterologous viral surface glycoproteins is not possible. Capsid transport and particle budding are driven by a highly specific interaction between the N-terminus of FV Gag and its cognate Elp domain [68], and even the F17/951 FFV serotype or the related PFV Env were much less active than the homologous FUV Env in the background of env‑deficient FFV-FUV-derived vectors [30]. A recent paper by Ho *et al.* [69] describes an adaptor system that allows the production of recombinant FV using fusion proteins containing a heterodimerization domain (HDD). These particles can be pseudotyped using a heterologous Env membrane-targeting domain (MTD) fusion protein. This approach could be promising for future targeted therapy approaches using FV vectors.

The efficacy of a given vector production system does not only depend on the different cis- and trans-acting vector components, but also on interactions between the transcription units used to express them. In FFV, promoter interference, representing competition for limited resources by identical (in this case, CMV-IE) or closely related promoters, may significantly decrease vector titers [54]. 

Efforts have been made to identify the cellular receptor for FV, which would allow clearer definition of FV tropism. Heparan sulfate has been identified as a possible co-receptor [70]. So far, no definitive main receptor has been identified, though the broad tropism of FV *in vitro* and *in vivo* suggests that it is a ubiquitous molecule or family of molecules.

## 6. Future Developments

The applicability and efficacy of RC and RD FFV-derived vectors have clearly been shown in different studies. While RD PFV-based vectors appear to infect a significantly broader range of human cells compared to FFV-based RD vectors [11], the advantage of the RC FFV vector system is its direct applicability to a fully permissive animal system, the outbred and immunocompetent cat. Here, issues concerning vector safety and vector targeting *in vivo* can be directly and easily addressed. In addition, the concept of RC vaccine vectors based on a non‑pathogenic and highly efficient transfer system for persistent diseases is of particular value. Recent data from HIV vaccine development have clearly shown that long-term and persistent exposition towards neutralization-relevant epitopes is essential for the induction and maturation of broadly neutralizing antibodies [71,72,73]. Therefore, persistently replicating vaccine vectors known to induce humoral and cellular immune responses and stably expressing the relevant epitopes over an extended period of time may be a rational and attractive choice for antigen expression. Here, FFV is the vector of choice, as both prerequisites to develop a lentivirus vaccine model are fulfilled: a well‑established and manageable animal model and an authentic challenge virus, feline immunodeficiency virus (FIV), a counterpart to HIV [25,74]. In this animal model, immunogenic protein domains or even complete functional proteins may be inserted into RC FFV vectors without destroying the viability of the FFV vector. Such chimeric RC vectors may have the capacity to induce long-term expression of the lentivirus (FIV)-derived vaccine antigens to establish protective immunity. In addition, systemic or mucosal administration of the engineered RC FFV vaccine vectors may lead to a generalized or local mucosal immunity capable of inhibiting or reducing primary infections. 

Due to the applicability and genetic manipulability of the cat model in the study and development of viral vectors [24], we will intensify our efforts in studying FFV vector-based gene and antigen transfer in cats. These studies will not only address issues of vector safety and applicability, but also those related to virus tropism in challenged and vector-treated animals. This is of prime importance for the entire FV field, since one of the current shortcomings of FV-based vectors is vector targeting (or unwanted secondary spread) within the treated patient, especially as *ex vivo* studies currently being performed, like those in dogs [75], may be eventually replaced by an *in vivo* transduction regimen. 

Though vaccination with whole virus particles may generate a more robust immune response, subviral or virus-like particles present an even safer alternative. The processes of particle assembly, budding and release of FVs differ greatly from those of orthoretroviruses. Several labs are currently working on providing the field with a deeper understanding of these processes, which is critical to the development of novel FV-based antigen scaffolds. Recent work from our lab showed that engineered FFV Gag is capable of inducing Env-independent subviral particle budding [54] and engineered Env may for Env-only virus-like particles [76]. Protein antigens can be incorporated into a foamy virus structural protein and be used in therapeutic or prophylactic vaccination against the corresponding disease. The use of viral derivatives not only circumvents the need to produce a functional protein, but also reduces the risk of insertional mutagenesis, often the biggest safety hurdle for retrovirus-based vaccines.

Ongoing work in our lab and others seeks to use FFV vectors containing therapeutic epitopes for vaccination in cats as a proof-of-concept experiment. As cats are a smaller animal model than primates, FFV vectors that show statistically significant and promising therapeutic results could be further translated into the primate system using PFV and, potentially, into human therapy. The role of FFV itself in human therapy may be limited, due to lack of productive infection in humans, but this has not been completely explored. As the cellular receptor for foamy viruses remains unknown, it is not possible to predict tropism in all tissues. 

RC FFV vectors may be also applicable to humans, for instance, in cancer therapy aimed at induction or enhancement of tumor-specific immunity or oncolysis. Here, the limited replication capacity of FFV and the FFV-derived RC vectors in the distantly related human host is predicted to strongly restrict uncontrolled vector spread to non-tumorous tissues or even to other persons. Provided that sufficiently concentrated vector stocks can be achieved and given that vector replication and spread in the tumor tissue is not completely abrogated, this concept significantly increases biological safety and containment of the therapeutic RC FV vectors and, thus, represents another challenging application for FFV-based vectors. 
